# Simplification of dosimetry in ^90^Y-radioembolization therapy by dual planar images

**DOI:** 10.1186/s12885-022-10392-y

**Published:** 2022-12-08

**Authors:** Mohammad Abuqbeitah, Özgür Taylan Akdağ, Mustafa Demir, Sertaç Asa, Kerim Sönmezoğlu

**Affiliations:** Department of Nuclear Medicine, Istanbul University - Cerrahpasa, Cerrahpasa Faculty of Medicine, Istanbul, Turkey

**Keywords:** 90Y therapy, Dosimetry, LSF, Attenuation & scatter correction, Absorbed dose

## Abstract

**Aim:**

The purpose was to provide a practical and effective method for performing reliable ^90^Y dosimetry based on ^99m^Tc-MAA and SPEC/CT. The impact of scatter correction (SC) and attenuation correction (AC) on the injected ^90^Y activity, lung shunt fraction (LSF) and the delivered dose to lung and liver compartments was investigated within the scope of the study.

**Material and methods:**

Eighteen eligible patients (F: 3, M: 15) were subjected to ^90^Y therapy. ^99m^Tc-MAA (111-222 MBq) was injected into the targeted liver, followed by whole-body scan (WBS) with peak-window at 140 keV (15% width) and one down-scatter window. SPECT/CT scan was subsequently acquired encompassing lung and liver regions. The LSFs were fashioned from standard WBS LSFwb (St), scatter corrected WBS LSFwb (Sc), only scatter corrected SPECT LSFspect (NoAC-SC) and SPECT/CT with attenuation and scatter correction LSFspect (AC-SC). The absorbed doses that would be delivered to tumor and injected healthy liver were estimated using different calculation modes involving AC-SC (SPECT/CT), NoAC-SC (SPECT), NoAC-NoSC+LSFwb (SC), AC-SC + LSFwb (St), and NoAC-NoSC+LSFwb (St).

**Results:**

The average deviations (range) in LSF values between standard LSFwb (St) and those from SPECT/CT (AC-SC), SPECT (NoAC-SC), and LSFwb (SC) were − 50% (− 29/− 71), − 32% (− 8/− 67), and − 45% (− 13/80), respectively. The suggested ^90^Y activity (GBq/Gy) was decreased within a range of 2-11%, 1-9%, and 2-7% by using LSFspect (AC-SC), LSFspect (NoAC-SC), and LSFwb (SC), respectively. Overall, two-sample t-test yielded no statistically significant difference (*p* < 0.05) in the absorbed doses to tumor and injected healthy liver between AC-SC (SPECT) and the rest of approaches with/and without AC and SC. However, a statistically significant difference (*p* < 0.05) was demonstrated in the lung shunt fractions and lung doses due to AC and SC. The LSFs from scatter corrected planar images LSFwb (SC) exhibited well agreement (R_2_ = 0.92) with SPECT/CT (AC-SC) and there was no statistically significant difference (*P*_value_ > 0.05) between both methods.

**Conclusion:**

It was deduced that SPECT/CT with attenuation and scatter correction plays a crucial role in the measurements of lung shunt fraction and dose as well as the total number of ^90^Y treatments. However, the absorbed dose to tumors and injected healthy liver was minimally affected by AC and SC. Besides, a good agreement was observed between LSF datasets from SPECT/CT versus scatter corrected WBS that can be alternatively and effectively used in ^90^Y dosimetry.

## Introduction

Hepatocellular carcinomas and secondary liver metastases are widespread disorders with potential liver failure in the vast majority of patients. Unfortunately, a small percentage of patients are eligible for curative therapy like resection and liver transplantation [1]. While, the inoperable cases undergo well-established palliative treatments including chemoembolization, radiofrequency, and yttrium-90 (^90^Y) radioembolization [2].

^90^Y-radioembolization is widely applied for the treatment of hepatocellular carcinomas and liver metastases by injecting micron-sized embolic particles loaded with ^90^Y via percutaneous intra-arterial techniques [3]. The loco-regional injection of radioactivity enables delivering high radiation doses to the tumor and limited dose to the normal tissue. ^90^Y activity decays by beta emission with mean energy of 932 keV, while no significant electromagnetic radiation is emitted except yielding small annihilation photons that enables ^90^Y positron emission tomography (PET) scan [4]. Therefore, technetium- albumin aggregated ^99m^Tc-MAA is alternatively used to surrogate ^90^Y microspheres and tailor the injected activity for therapy.

Scintigraphy imaging is usually performed within one half to 1 h after ^99m^Tc-MAA injection revealing the distribution and leakage of the injected radioactivity [5]. The classic imaging protocol invariably includes whole-body scan, and single photon computed tomography SPECT scan to recognize tumor and non-tumor regions. More recently, SPECT integrated with computed tomography CT has been extensively used with definite anatomical information and attenuation correction. The toxicity risk of ^90^Y therapy is inseparably associated with microspheres leakage to lungs and the surrounding healthy liver. Conventionally, the standard lung shunt fraction is computed from ^99m^Tc whole-body scan, while SPECT scan is acquired to thoroughly quantify the tumor and injected healthy liver IHL partitions [6]. However, the occurring attenuation across the body thickness and scatter radiation might significantly affect the dose prediction and ^90^Y-activity assignment. It is well known that the true events are properly caught when the emitted gamma rays perpendicularly pass from the collimator and interact with the NaI (Tl) crystal by photoelectron effect. However, the compton interaction with the crystal yields scatter events and the scattered photons might further undergo subsequent interactions in the crystal/or completely escape from the detector. Additionally, the object scattering in the entire organs has a certain contribution that might be recorded as true events. Ultimately, a pulse height analyser (PHA) is operated to reject the scatter events, however, it might fail to totally reject these events and allow acceptance in false locations. Besides, the energy of the scattered rays partially decreases generating a weak signal that is finally filtered by the energy discriminator. However, scatter events with small scattering angle might be also accepted as true with a shifted position up to few centimetres from the origin of emission [7].

To this end, the potential effect of scatter correction SC and attenuation correction AC on lung shunt fraction LSF has been limitedly explored in ^90^Y radioembolization dosimetry. This study aimed to elucidate the impact of AC and SC on the lung dose and injected healthy liver dose in addition to provide a simplified approach for ^90^Y dosimetry with comparable performance to SPECT/CT as gold standard.

## Methodology

**Fig. 1:**
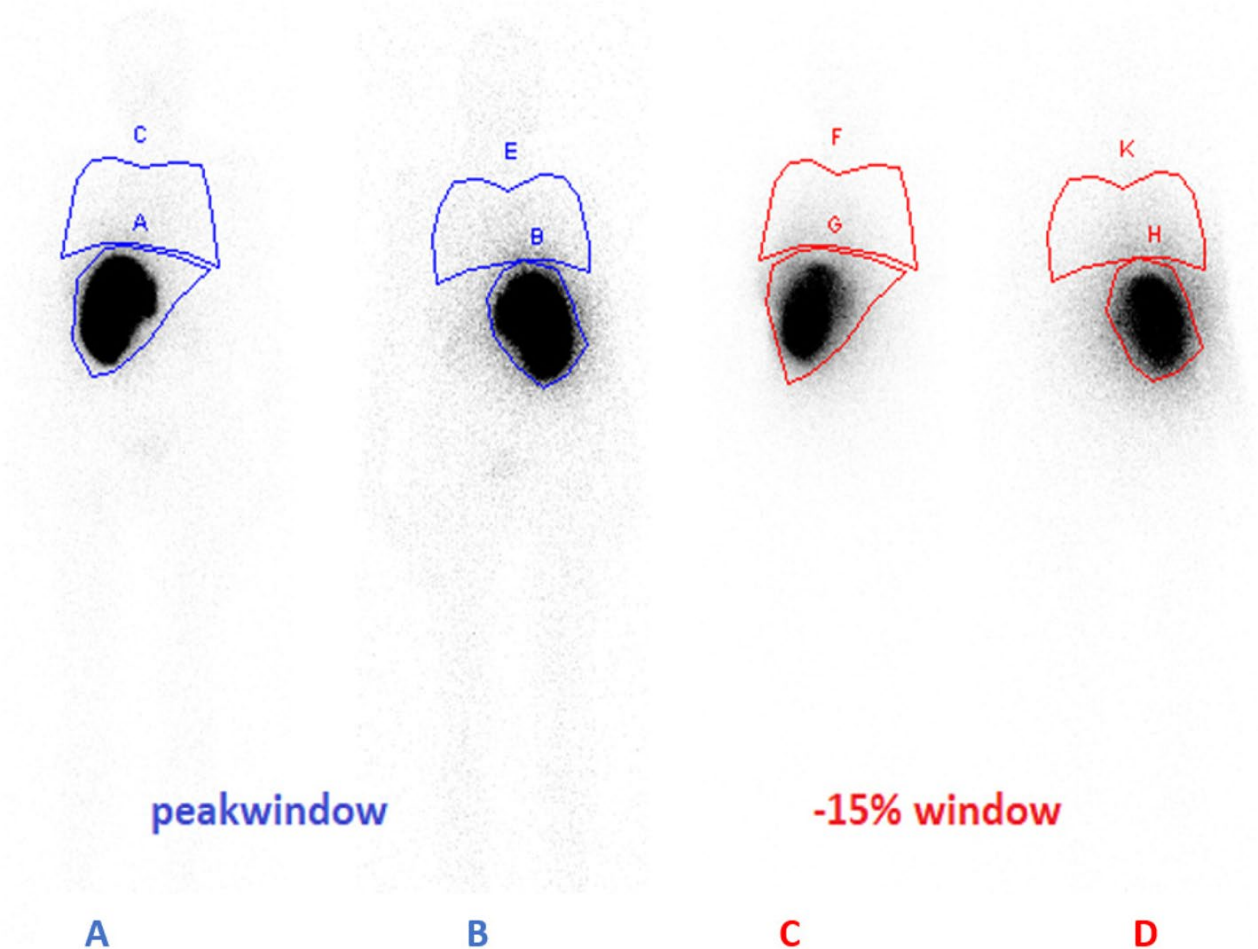
Dual planar images of a patient injected by 99mTc-MAA; (**A** + **B**): anterior and posterior images of the peak window; (**C** + **D**): anterior and posterior images of the scatter window

Eighteen patients (F: 3, M: 15) (8: colon Ca, 2: HCC, others: 8) were included in the current study and assigned consent form was obtained from each participant. A range of 111-222 MBq ^99m^Tc-MAA was intra-arterially injected in the targeted liver at the interventional radiology department of Istanbul University-Cerrahpasa. After injection, whole-body scan (WBS) was acquired with adjusted settings includes peak-window at 140 keV (15% width), and one down-scatter window (15% width). A dual-headed gamma camera (Symbia™ T Series SPECT/CT) was used for imaging. According to this protocol, two planar images were created for each patient, as seen in Fig. 1. Region of interest ROI was delineated on the lungs and liver organs separately and the derived counts were used to calculate lung shunt fraction. The LSF was calculated from the peak-window image by:

1 $$\textrm{LF}=\frac{\sqrt{La\times Lp}}{\left(\sqrt{La\times Lp}+\sqrt{Lva\times Lvp}\right)}$$

La) lungs anterior counts. Lp) lungs posterior counts. Lva) liver anterior counts. Lvp) liver posterior counts.

Then, scatter corrected LSF was obtained and symbolized as LSFwb (SC) after applying scatter correction over the planar images by dual-energy windows (±15%) as follows:

2 $$\textrm{Ctrue}=\textrm{Cmain}-\left[\frac{Clow}{Wlow}\right]\ \frac{Wmain}{2}$$

C_true_: organ’s scatter corrected counts, C_main_: counts frmom peak window image. C_low_: counts from down-scatter window image, W_low_: scatter-window fraction. W_main_: peak-window fraction.

SPECT/CT scan was instantly conducted after scintigraphy encompassing lungs and liver regions. The windows settings were similar to those used in planar imaging and 64 projections were acquired with 25 seconds/projection. Scatter correction was carried out similarly by dual-energy window (15%). Iteration method of ordered subsets expectation maximization (OSEM) was applied for reconstruction with 10 iterations and 8 subsets, pursued by low pass filtering (Gaussian 9 mm) for noise suppression. The reconstruction was repeated to generate three types of SPECT images: first, SPECT images with attenuation and pixel-wise scatter correction; coded as (AC-SC), second: SPECT images with no attenuation and no scatter correction; coded as (NoAC-NoSC), and third: SPECT image with only scatter correction; coded as (NoAC-SC).

**Fig. 2:**
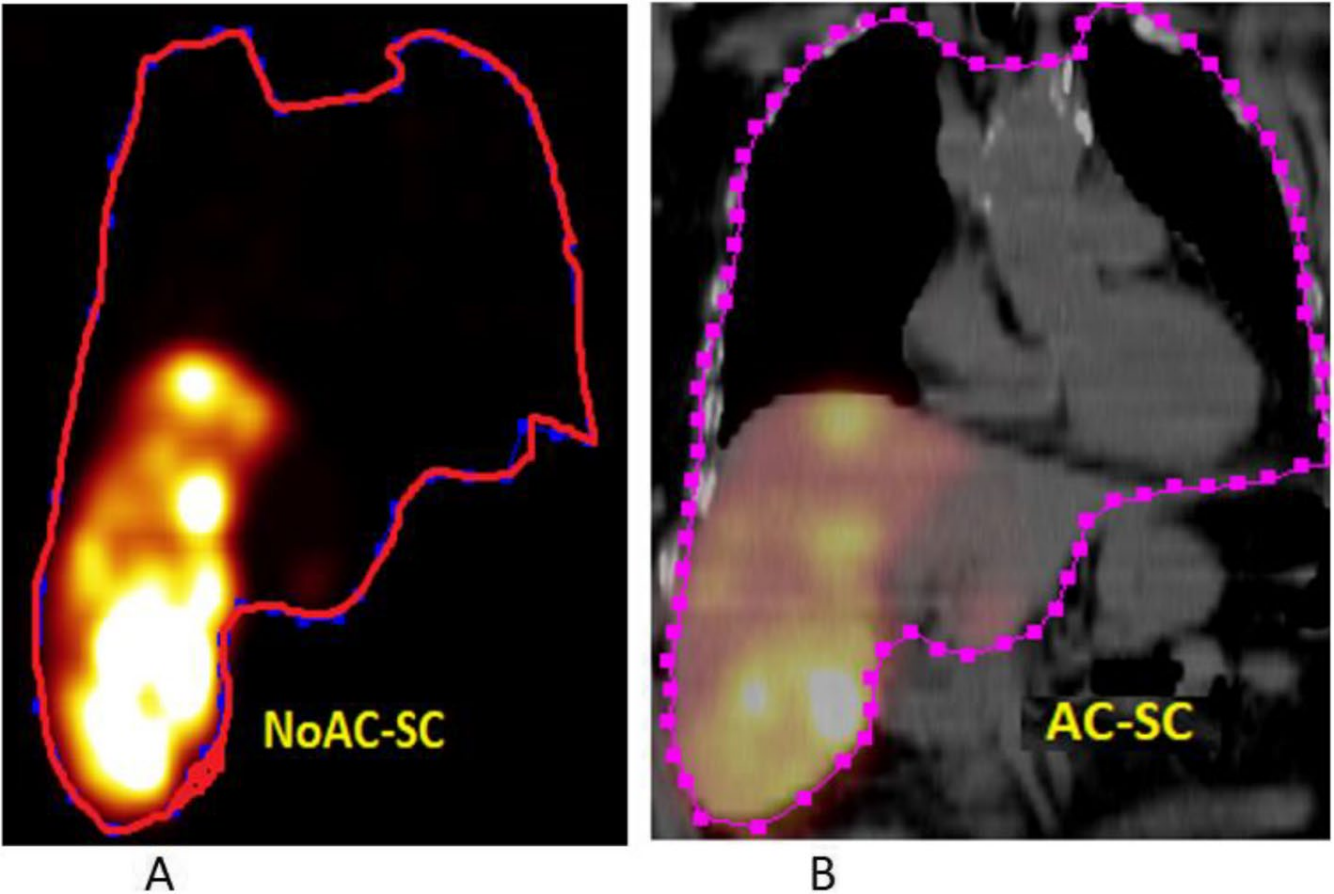
Region of interests (ROIs) delineation for lungs and liver using freehand drawing contours over (**A**) only scatter correct SPECT images NoAC-SC, and (**B**) scatter and attenuation corrected SPECT images AC-SC

**Fig. 3:**
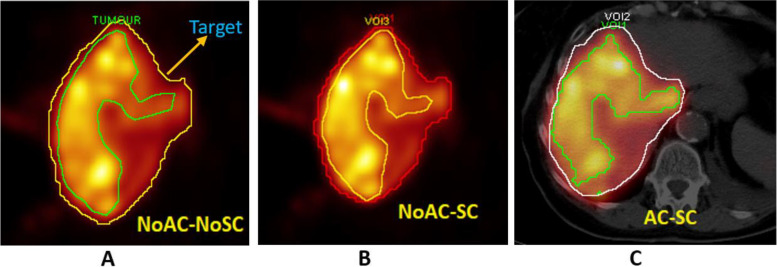
**A** ROIs (region of interests) delineation over the target and tumor volumes using automatic iso-contour tool for SPECT images with (**A**): no scatter correction and no attenuation correction NoAC-NoSC, (**B**): only scatter correction NoAC-SC, and (**C**): both scatter correction and attenuation correction AC-SC

**Fig. 4:**
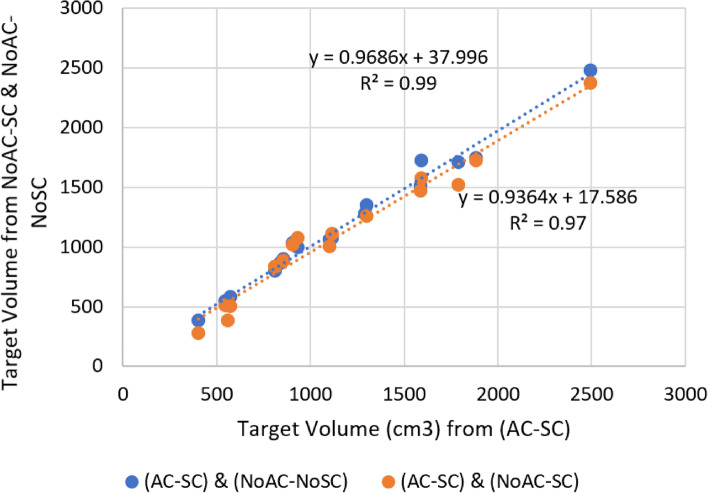
The agreement between target volumes obtained from SPECT images with attenuation and scatter correction AC-SC, only scatter correction NoAC-SC, and neither scatter correction nor attenuation correction NoAC-NoSC

The segmentation of liver and lungs was performed over the CT images (Fig. 2) by freehand drawing contours. The injected healthy liver and tumor regions were segmented on the SPECT images via iso-contour tool (Fig. 3) based on an intensity threshold by region growing. The generated volume and counts were displayed on a desktop computer terminal for each organ and compartment. The IHL volume was compared between different SPECT-image types as seen in Fig. 4, to be next used in the suggested activity and lung dose calculation.

An experienced ^90^Y dosimetrist and physician were cooperated in the registration and segmentation process. The lung shunt fractions were calculated from (AC-SC), and (NoAC-SC) SPECT images by the below equation:

3 $${\textrm{LSF}}_{\textrm{spect}}:\textrm{Lc}/\left(\textrm{Lc}+\textrm{LVc}\right)$$

LSF _spect_: lung shunt fraction from SPECT images, Lc: lungscount, LVc: liver counts.

The lung shunt fractions were calculated by different modules involving: a- LSFwb (St): from the standard whole-body scan, b- LSFwb (SC): from scatter corrected whole-body scan; c- LSFspect (AC-SC): from SPECT/CT with attenuation and scatter correction; and d- LSFspect (NoAC-SC): from SPECT with only scatter correction.

Lung dose was calculated by the following equation:

4 $$\textrm{D}\ \left(\textrm{Gy}\right)=\textrm{A}\ \left(\textrm{GBq}\right)\times 49.33/\textrm{M}\ \left(\textrm{Kg}\right)$$

D: dose, A: activity, M: mass.

The absorbed dose to tumor and injected healthy liver was estimated using the following approaches:

1. AC-SC (SPECT/CT): lung shunt fraction, tumor and target quantification were all made by SPECT/CT images with AC and SC.
2. NoAC-SC (SPECT): lung shunt fractions, tumor and target quantification were all obtained from scatter corrected SPECT images.
3. AC-SC + LSFwb (St) lung shunt fractions were computed from the standard WBS, while the tumor and target quantification was made from SPECT/CT (AC-SC).
4. NoAC-NoSC+LSFwb (SC) lung shunt fractions were calculated from scatter corrected whole-body scans, while the tumor and target quantification were made from SPECT images (NoAC-NoSC).
5. NoAC-NoSC+LSFwb (St): lung shunt fractions were calculated from the standard WBS, while the tumor and target quantification was from NoAC-NoSC SPECT images.

MIRD scheme was used in the dose calculation to tumor and non-tumor partitions as follows [8]:

5 $$\textrm{Liver}\ \textrm{uptake}=\left(1- SF\right)\left[\frac{m_{liver}}{\left({m}_{tumour}\times TLR\right)+{m}_{liver}}\right]$$

Activity to be administered for a certain absorbed dose:

6 $$\textrm{Activityadmin}\ \left(\textrm{mCi}\right)=\frac{{\textrm{dose}}_{\textrm{liver}}\ \left(\textrm{rad}\right)\times {\textrm{m}}_{\textrm{liver}}\ \left(\textrm{gm}\right)}{\textrm{184,000}\times \textrm{liver}\ \textrm{fractional}\ \textrm{uptake}\ }$$

7 $$\textrm{Tumor}\ \textrm{uptake}=\left(1-\textrm{SF}\right)\left[\frac{\textrm{TLR}\times {\textrm{m}}_{\textrm{tumor}}}{\left({\textrm{m}}_{\textrm{tumor}}\times \textrm{TLR}\right)+{\textrm{m}}_{\textrm{liver}}}\right]$$

8 $${\textrm{Dose}}_{\textrm{tumor}}\left(\textrm{rad}\right)=\frac{{\textrm{Activity}}_{\textrm{total}}\left(\textrm{mCi}\right)\times 184000\times {\textrm{UPTAKE}}_{\textrm{tumor}}}{{\textrm{m}}_{\textrm{tumor}}\left(\textrm{g}\right)}$$

### Statistical analysis

The statistical analysis was performed via IBM/SPSS statistics 20 software. Two-sample t-test was used to compare the paired calculation methods in LSF, suggested ^90^Y activity and absorbed dose to tumor and IHL.

## Results

**Fig. 5:**
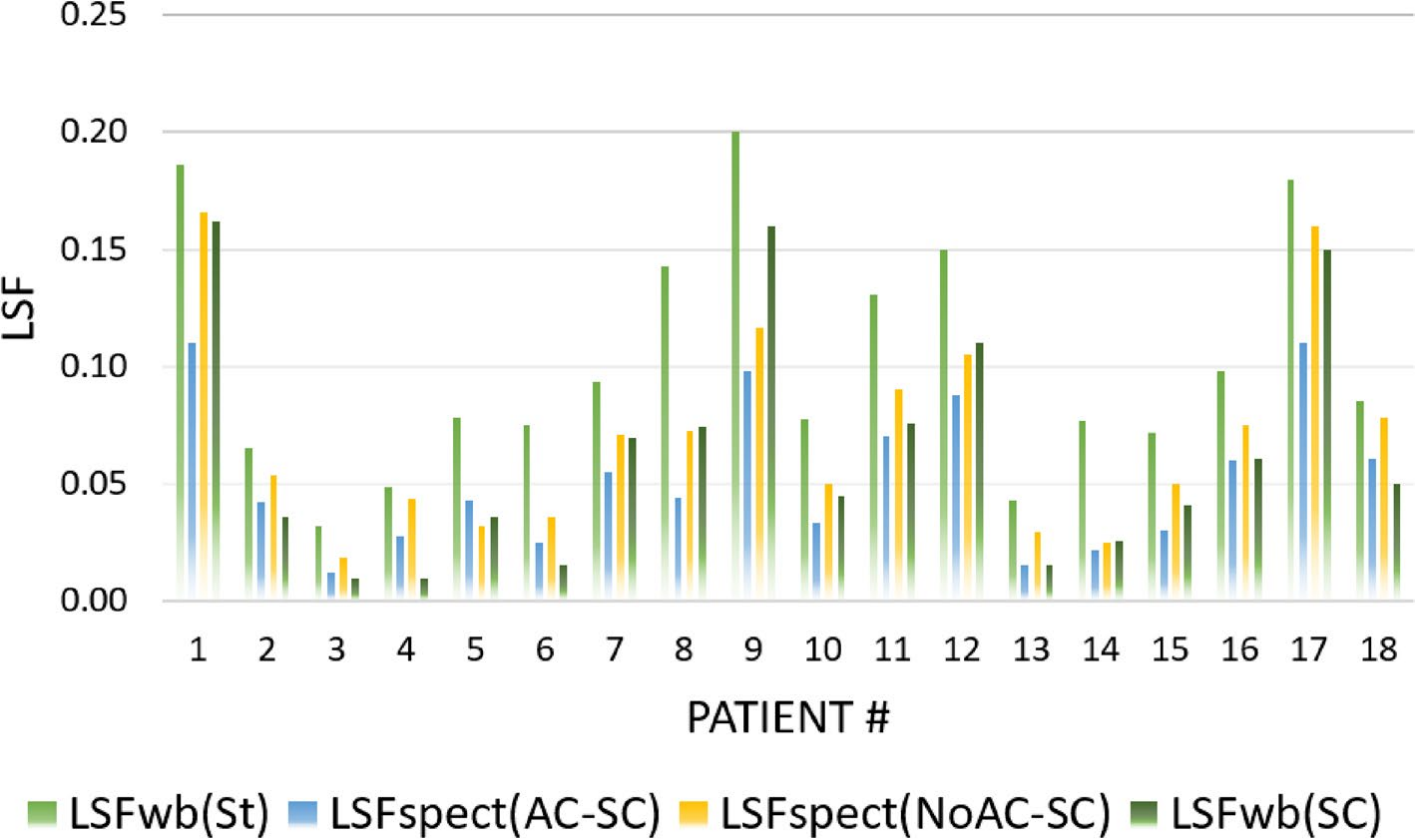
The patients corresponding LSF values from different models including LSFwb (St) from standard whole-body scan, LSFwb (SC) from scatter corrected whole-body scan, LSFspect (AC-SC) from SPECT/CT with attenuation and scatter correction, and LSFspect (NoAC-SC) from SPECT with only scatter correction

**Fig. 6:**
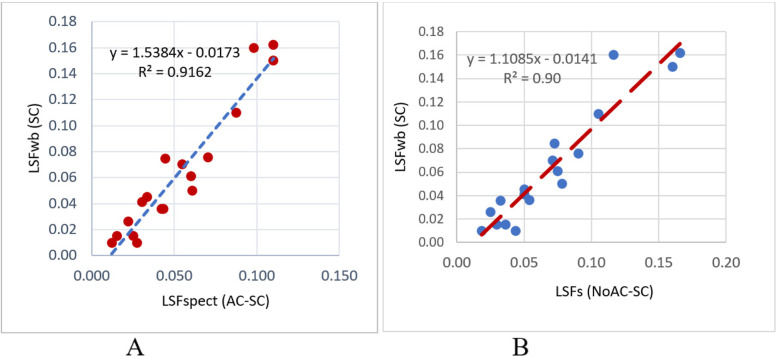
The agreement between LSFs from scatter corrected whole body scan LSFwb (SC) and models of LSFspect (AC-SC) from SPECT/CT with attenuation and scatter correction (**A**), and LSFspect (NoAC-SC) from SPECT with only scatter correction (**B**)

The lung shunt fractions of 18 patients underwent ^99m^Tc-MAA procedure were explicated in Table 1 and visually compared across different categories in the bar chart displayed in Fig. 5. In major comparison, the deviation in the calculated LSFs between WBS and other image formats were reported in Table 2. The obtained results indicated that lung shunt fractions from standard whole-body scan LSFwb (St) were considerably larger than SPECT/CT with attenuation and scatter correction (mean: 50%, range: 29-71%). A smaller deviation (mean − 32%) was shown between the LSFs from scatter corrected SPECT (NoAC-SC) and LSFwb (St). Whereas, the LSF values from scatter corrected whole-body scan LSFwb (SC) were analogous to SPECT/CT (AC-SC) with mean deviation of − 45% (range − 13/− 80). Figure 6 portrayed a well agreement (R_2_ = 0.92) between LSFs from scatter correct planar images LSFwb (SC) and SPECT images with AC and SC.

**Table 1:** Lung shunt fractions from different image formats; LSFwb (St) from standard whole-body scan, LSFwb (SC) from scatter corrected whole-body scan, LSFspect (AC-SC) from SPECT/CT with attenuation and scatter correction, and LSFspect (NoAC-SC) from SPECT with only scatter correction

No	LSFwb (St)	LSFspect (AC-SC)	LSFwb (SC)	LSFspect (NoAc-SC)
1	0.186	0.110	0.162	0.166
2	0.066	0.042	0.036	0.054
3	0.032	0.012	0.010	0.019
4	0.049	0.027	0.010	0.044
5	0.078	0.043	0.036	0.032
6	0.075	0.025	0.015	0.036
7	0.094	0.055	0.070	0.071
8	0.143	0.044	0.075	0.073
9	0.200	0.098	0.160	0.116
10	0.077	0.034	0.045	0.050
11	0.131	0.070	0.076	0.090
12	0.150	0.088	0.110	0.105
13	0.043	0.015	0.015	0.029
14	0.077	0.022	0.026	0.025
15	0.072	0.031	0.041	0.050
16	0.098	0.060	0.061	0.075
17	0.180	0.110	0.150	0.160
18	0.085	0.061	0.050	0.078
Mean	0.102	0.053	0.064	0.071
SD	0.049	0.031	0.049	0.042

**Table 2:** Deviation of LSFs from different images formats to standard whole-body scan LSFwb (St) including LSFwb (SC) from scatter corrected whole-body scan, LSFspect (AC-SC) from SPECT/CT with attenuation and scatter correction, and LSFspect (NoAC-SC) from SPECT with only scatter correction

Method	LSFspect (AC-SC)	LSFspect (NoAC-SC)	LSFwb (SC)
**Mean %**	−50	−32	−45
**Min/Max %**	−29/−71	−8/−67	− 13/−80
***P***_**value**_	0.002	0.049	0.016

Moreover, no statistically significant difference (*P*_value_ > 0.05) was found between LSFwb (SC) and SPECT (AC-SC), however, a significant difference was yielded between LSFwb (St) and the rest of the LSF calculation models (*P*_value_ < 0.05).

Since, a well agreement (R_2_ = 0.97) was demonstrated in the calculated target volumes from all of the SPECT images (Fig. 4), a fixed target volume (by SPECT with AC-SC) was employed to estimate the suggested activity and lung dose with changeable LSF. Consequently, a slight disparity (− 2 to − 11%) was obviated between the suggested activities from standard LSFwb (St) and the other calculation methods, as reported in Table 3. However, the variation was dramatically large in the lung doses with replacing the LSF calculation models. For instance, the computed lung doses from SPECT/CT (AC-SC) had the largest deviation to the LSFwb (St) with an average of − 53% (range: − 34/− 72%). Likewise, the lung doses from scatter corrected planar images showed a mean deviation of − 48% (range: − 18/− 72%), while the least deviation was in favour of the scatter corrected SPECT (NoAC-SC) with mean deviation of − 33% ranging from − 12% to − 64%. In comparison, there was no statistically significant difference (*P*_value_ > 0.05) in the suggested activity between LSFwb (St) and the more complex models, whereas a significant difference was evidently manifested in the lung absorbed doses (*p* < 0.05) amidst the standard LSFwb (St) and models of LSFwb (SC) and the most advanced one LSFspect (AC- SC), as displayed in Table 3.

**Table 3:** The deviation in the suggested activity and lung doses between standard whole body scan LSFwb (St) and models of LSFwb (SC): from scatter corrected whole-body scan, LSFspect (AC-SC): from SPECT/CT with attenuation and scatter correction, and LSFspect (NoAC-SC): from SPECT with only scatter correction

	LSFspect (AC-SC)	LSFspect (NoAC-SC)	LSFwb (SC)
**Suggested Activity**	Mean − 5.3%	Mean − 3.2%	Mean − 5.5%
	Range (−2/−11)	Range (−1/−9)	Range (− 2/− 7)
	*P*_value_ = 0.55	*P*_value_ = 0.58	*P*_value_ = 0.52
**Lung Dose**	Mean − 53%	Mean − 33%	Mean − 48%
	Range (− 34/− 72)	Range (−12/− 64)	Range (− 18/− 72)
	*P*_value_ = 0.02	*P*_value_ = 0.12	*P*_value_ = 0.03

**Fig. 7:**
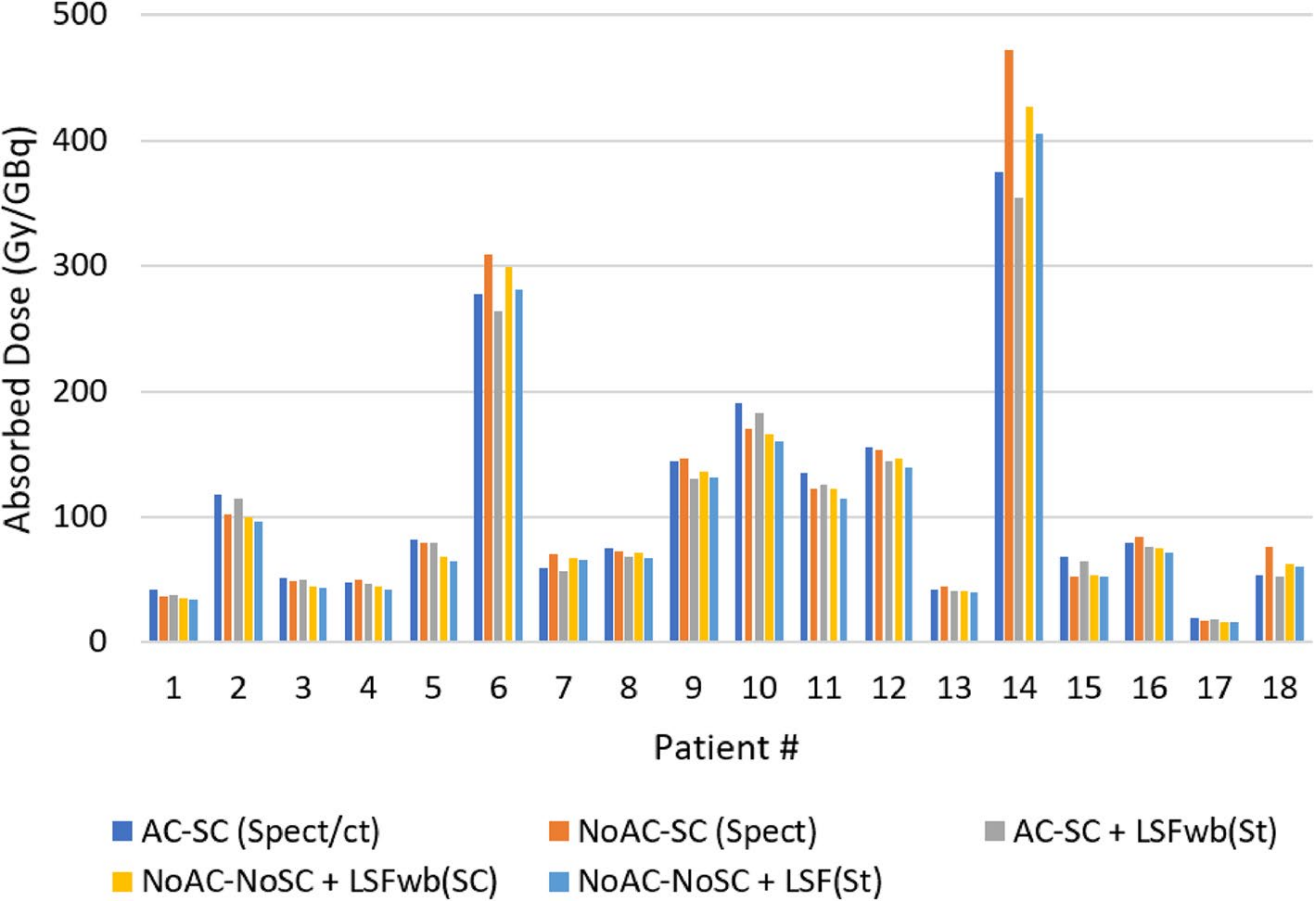
Absorbed dose to tumor (Gy/GBq) with different dosimetry approaches; AC-SC (SPECT/CT): lung shunt fraction, tumor and target quantification were made by SPECT/CT images with AC and SC, NoAC-SC (SPECT): lung shunt fractions, tumor and target quantification were obtained from scatter corrected SPECT images, AC-SC + LSFwb (St) lung shunt fractions were computed from the standard WBS, while the tumor and target quantification was made from SPECT/CT (AC-SC), NoAC-NoSC+LSFwb (SC) lung shunt fractions were calculated from scatter corrected whole-body scans, while the tumor and target quantification were made from SPECT images (NoAC-NoSC), and NoAC-NoSC+LSFwb (St): lung shunt fractions were calculated from the standard WBS, while the tumor and target quantification was from NoAC-NoSC SPECT images

**Fig. 8:**
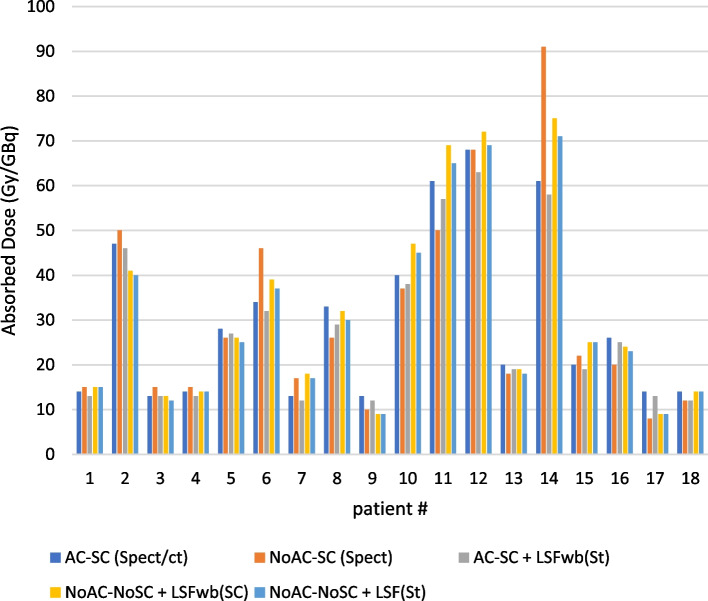
Absorbed dose (Gy/GBq) to IHL with different dosimetry approaches; AC-SC (SPECT/CT): lung shunt fraction, tumor and target quantification were made by SPECT/CT images with AC and SC, NoAC-SC (SPECT): lung shunt fractions, tumor and target quantification were obtained from scatter corrected SPECT images, AC-SC + LSFwb (St) lung shunt fractions were computed from the standard WBS, while the tumor and target quantification was made from SPECT/CT (AC-SC), NoAC-NoSC+LSFwb (SC) lung shunt fractions were calculated from scatter corrected whole-body scans, while the tumor and target quantification were made from SPECT images (NoAC-NoSC), and NoAC-NoSC+LSFwb (St): lung shunt fractions were calculated from the standard WBS, while the tumor and target quantification was from NoAC-NoSC SPECT images

On the other hand, as stated earlier, different calculation modes were employed to anticipate the radiation dose to tumors and injected healthy liver. Overall, the two-sample t test (Mann-Whitney t test) yielded no statistically significant difference (p < 0.05) in tumor and IHL absorbed dose between datasets from (NoAC-SC (SPECT), NoAC-NoSC+LSFwb (SC), AC-SC + LSFwb (St), and NoAC-NoSC+LSFwb (St)) versus *AC-SC (SPECT/CT)*. For more elaboration, an interpatient comparison was made showing the difference in the absorbed doses to liver components from the aforementioned dosimetry approaches. Figure 7 depicted the variation in the absorbed dose to tumor and likewise the absorbed doses to the IHL were displayed in Fig. 8 on patient-specific basis.

## Discussion

^99m^Tc-MAA has been widely used as a surrogate for ^90^Y labelled microspheres regardless of the outstanding argumentation on the non-uniform distribution. An approximately 0.5 × 10E6 MAA particles labelled with ^99m^Tc are typically injected into the hepatic artery, while about 2 × 10E6 microspheres are found in 1.5 GBq ^90^Y [9]. The assessment of lung shunt fraction in ^90^Y therapy is routinely made depending on post-injection scintigraphy and the activity to administer is prescribed accordingly.

Nowadays, individualized treatment is preferentially followed since it enables effective dose delivery to tumor and sparing healthy tissues. In ^90^Y dosimetry, lung and liver parenchyma are the main organs at risk that must remain within tolerable dose limits. On hand, the dose restrictions of the healthy liver have been updated with respect to normal tissue complication probability (NTCP) factors. For example, dose limit of 70 Gy to liver parenchyma is corresponding to an approximately 15% NTCP, however, increasing the absorbed dose to as high as 70-105 Gy raises the liver decompensation to 40% [10].

On the other hand, lung shunt fractions were originally derived from ^99m^Tc planar imaging (whole-body scan) and the single-treatment lung dose was limited to 30 Gy [11]. In resin microsphere, the whole activity is safely injected to the patients with LSF < 10%, while ^90^Y therapy is contraindicated with LSFs exceeding 20%. Moreover, the injected activity is reduced by 40% for patients with LSF between 15 and 20%, and down to 20% in case of 10-15% LSF range [12]. In this respect, scatter radiation and overlying tissue attenuation remain crucial issues and key-factors in image based quantification. In particular, scatter radiation substantially degrades image contrast by adding counts to both the background and the true signal. Thus, the quantification degrading factors were investigated within the scope of this work. In result, it was revealed that the variation in lung shunt fractions between the standard WBS and SPECT/CT (AC-SC) ranged from 28 to 71% with an average of 50%. This variation has great impact on the lung dose estimation and ^90^Y therapies. More importantly, well-validated LSFs might be reasonable justification for those patients ‘excluded’ from ^90^Y therapy due to LSF ≥22%. Also, more ^90^Y therapy sessions can be safely held taking into account the cumulative dose limit to lung ≈ 50 Gy [11]. For instance, one patient with 33% LSF from standard WBS, was injected by 0.99 GBq ^90^Y according to the LSF (21%) from SPECT/CT with (AC-SC). The mean absorbed dose to tumor and IHL was 165 Gy and 74 Gy, while the corresponding lung dose was 10 Gy. In consequence, no toxicity indications have been manifested over 3 months of patient follow-up.

On the other hand, no statistically significant difference was found in the absorbed dose to tumor and IHL in addition to the suggested activity between datasets obtained from different approaches (Figs. 7 and 8) versus AC-SC (SPECT/CT). In comparison, Gallio et al. reported that NoAC-SC images led to amount of activity not significantly different from the reference AC-SC images; while AC-NoSC and NoAC-NoSC images yielded significantly different activity prescriptions [13]. Similar conclusions were reported in a study with voxel-wise analysis [14]. Likewise, our study indicated no statistically significant difference (*p*_value_ < 0.05) in the suggested activities from NoAC-SC and the reference AC-SC at the organ level. However, the lung shunt fractions and the resultant lung doses are substantially affected by AC and SC. Allred and co-authors reported a liver/lung torso phantom study demonstrating improved accuracy in LSF estimation based on SPECT/CT with AC and SC compared with planar imaging (up to 44% overestimation). It was also stated that no statistically significant difference was found between LSFs from ^99m^Tc-MAA SPECT/CT and ^90^Y PET/CT, while a poor correlation (R_2_ = 0.44) was found between the planar and SPECT/CT based LSFs [15]. This leads to draw an inference that LSFs from scatter corercted WBS would have similarly strong association with ^90^Y- PET/CT.

A limitation to be addressed is the misalignment between SPECT and CT images due to respiratory motion that enhances statistical errors in the organs true counts. One technique has been recently described that helps to capture the mis-registered signals due to SPECT and CT different resolutions, and to minimize the impact of liver shine-through into the right lung contours by: 1-expanding the liver contours for a couple of centimeters (e.g 2 cm), 2- Deriving left lung counts per unit volume 3- multiplying the total lung volume by the derived count/cm^3^ factor [16]. Accordingly, the uncertainties attributable to variability in contouring, LSFs, and Lung doses were estimated as 9, 10, and 13%, respectively. Meanwhile, the Lung shunt fractions and dose measurements were reported lower than planar LSF with average of 63 and 53%, respectively [16]. The current work lacks technical accommodation for shine effect, however, the proposed results were plausibly consistent with that study involving shine-effect correction [16].

From the dosimetry viewpoint, the partitioning method is based on segmentation of tumor and non-tumor regions, and the computed absorbed doses are attributed to an entire region. However, the dose distribution throughout the object volume is heterogynous due to liver micro-vessels structure and long β particles range that might escape farther and alter the deposited energy in the selected ROIs. Thus, tracking energy transport from clusters of point-sources emitting beta radiation is considered the most accurate route for achieving super dosimetry, yet this method is out of use in the clinical practice [17]. Otherwise, it is possible to optimise the administered ^90^Y activity based upon well- validated LSFs and IHL doses taking into account fixed execution of SC and AC when possible or alternative scenarios with high precision, in addition, to update absolute dose limits to the critical organs in ^90^Y-radioembolization.

To this end, the effect of SC and AC saliently predominates in lung shunt fractions and doses. The technical limitations in SPECT/CT boost resorting to reliable methods in the daily practice with comparable accuracy and practical indications like scatter corrected planar images method.

## Conclusion

This study emphasized that AC and SC extremely influence LSF and lung dose, while there much less impact on the absorbed dose to tumor and injected healthy liver in 90Y therapy. In addition, a good agreement was observed between LSF datasets from SPECT/CT versus scatter corrected WBS supporting the potential to adopt cost-effective dosimetry method based on dual planar images for 90Y therapy planning.

## Data Availability

The datasets used and/or analysed during the current study are available from the corresponding author on reasonable request.
